# Estrogens and breast cancer

**DOI:** 10.1016/j.annonc.2024.10.824

**Published:** 2024-11-08

**Authors:** J. Kim, P. N. Munster

**Affiliations:** 1 Department of Biochemistry and Molecular Biology, Indiana University School of Medicine, Indiana University Melvin and Bren Simon Comprehensive Cancer Center, Indianapolis;; 2 Department of Medicine, Center for BRCA Research, UCSF Helen Diller Family Comprehensive Cancer Center, University of California, San Francisco, USA

**Keywords:** estrogen, progesterone, progestin, progestogen, breast cancer, hormone replacement therapy

## Abstract

Estrogens have been associated with an increase in breast cancer risk. Yet emerging clinical and experimental evidence points to progestogens [endogenous progesterone or synthetic progesterone (progestin)] as the primary hormonal driver underlying seemingly estrogen-associated breast cancer risk. Estrogens may contribute to breast cancer risk indirectly by induction of the progesterone receptor and thus amplifying progesterone signaling. Large studies of hormonal contraceptives suggest that the small increase in breast cancer risk from hormonal contraceptives is mainly attributable to progestins, not estrogens. Estrogen-plus-progestin hormone replacement therapy (HRT) has consistently shown an increase in breast cancer risk among postmenopausal women, whereas estrogen-alone HRT has little impact on breast cancer risk in naturally or surgically menopausal women. In particular, the long-term follow-up of the Women’s Health Initiative (WHI) randomized trials suggests a benefit of estrogen alone. Recent data further indicate that endogenously elevated estrogen during assisted reproductive technology (ART) exhibits little adverse effect on or potentially a reduction in breast cancer risk and recurrence. Also, accumulating evidence suggests that inhibition of progesterone signaling is a critical mechanism underlying the risk-reducing and therapeutic effects of antiestrogens. Estrogen HRT has shown an array of proven benefits, including ameliorating menopausal symptoms and improving bone health. Collective evidence thus suggests that estrogen HRT is likely to offer health benefits to perimenopausal or postmenopausal women, including breast cancer survivors, as well as young *BRCA1/2* carriers with prophylactic oophorectomy for ovarian cancer prevention.

Estrogen and progesterone are principal hormones in a woman’s life. Before menopause, women are exposed to regularly varying levels of endogenous estrogens and progesterone released during repeated menstrual cycles and potentially to more sustained levels of these hormones during pregnancy. Reproductive-age women may be further exposed to these hormones via oral contraceptives, composed of a progestin with or without an estrogen, and other forms of hormonal contraception.^1^ Near or after menopause, hormone replacement therapy (HRT)—estrogen alone or combined with a progestin—may be offered to mitigate menopausal symptoms arising from precipitous declines in endogenous estrogen production and to reduce the risk of bone fractures.^2–5^ Women with prior use of oral contraceptive pills show a significantly reduced risk of ovarian cancer and endometrial cancer.^6,7^ Despite the intended medical effects and additional health benefits, these hormonal agents, especially estrogens, remain a lingering concern for breast cancer risk.^8–11^

In this review, we have aimed to comprehensively evaluate the association between estrogens and breast cancer. Given the expansive scope of scientific investigations pertaining to this topic, we focus on findings from randomized clinical trials (RCTs), prospective cohort studies, and meta-analyses with rigor and a large sample size, as well as preclinical mechanistic studies. To be inclusive on the topic with depth, we examined vast evidence, via extensive search on PubMed, from clinical and basic experimental studies, which include hormonal contraceptives, HRT, estrogen-elevating fertility treatments such as *in vitro* fertilization (IVF), reproductive factors, steroid hormone synthesis and metabolism, breast cancer therapies, basic experimental studies, and animal research. Ultimately, we have sought to connect the dots by critically assessing and cohesively integrating wide-ranging, oft-incongruent findings into the conclusions based on the totality of current evidence.

Emerging from this comprehensive review is a new perspective on the role of hormones in breast cancer risk. What initially appeared to be estrogen-associated breast cancer risk may actually be driven by progestogens. Emerging evidence suggests that progestins, not estrogens, from hormonal contraceptives are most likely responsible for the small increase in breast cancer risk. Combined evidence suggests that progestogens are likely the primary oncogenic hormonal factor that underlies the increased breast cancer risk associated with estrogen-containing HRT, while estrogens may contribute to breast cancer risk by augmenting progesterone signaling. Additionally, suppression of progesterone signaling may be a key mechanism underlying the risk-reducing and therapeutic effects of antiestrogen breast cancer therapies. Also, vastly elevated levels of endogenous estrogens induced by fertility treatments, such as IVF/ART, appear to have no adverse effect on breast cancer risk or recurrence. Collective evidence suggests that estrogen therapy, coupled with minimal use of a progestogen, might be a safe option for women in need of HRT.

## ENDOGENOUS HORMONE PRODUCTION

During the menstrual cycle, estradiol (E_2_) is the predominant ovarian estrogen produced by ovarian follicles and the corpus luteum. E_2_ levels gradually increase from 20–50 pg/ml to 50–80 pg/ml during the early- to mid-follicular phase, rapidly rise to peak at ~250 pg/ml (130–400 pg/ml) before the luteinizing hormone (LH) surge, and remain around 120 pg/ml (<20–241 pg/ml) during the luteal phase before dropping to 30–50 pg/ml during menses.^12–16^ Estrone (E_1_) levels vary from <15 to ~200 pg/ml^14–16^ and estriol (E_3_) levels remain low yet steady at 7–11 pg/ml during the menstrual cycle.^17^ Menstrual progesterone levels are low at <1.5 ng/ml before the LH surge and rapidly rise to 10–20 ng/ml in the luteal phase.^12,16^ During pregnancy, progesterone is produced by the corpus luteum of the ovary for the first 6–8 weeks of pregnancy and thereafter by the placenta for the remainder of pregnancy ranging from 10 to 40 ng/ml in the first trimester and progressively increasing to reach 150–175 ng/ml at term.^13,18–21^ In addition to high levels of E_2_ during pregnancy (200–20 000 pg/ml), E_3_ becomes the major estrogen produced by the placenta to reach as high as 8000–13 000 pg/ml at term.^18–22^ E_1_ levels are also progressively elevated, ranging from <1000 pg/ml in the first trimester to 6000–11 000 pg/ml at term.^18,21,22^

Even after menopause, women continue to produce low levels of estrogens and progesterone (<0.5 ng/ml).^13,23^ Postmenopausal estrogens consist of E_1_ (25–42 pg/ml), the dominant estrogen after menopause, and also E_2_ (10–25 pg/ml).^24,25^ E_3_ levels remain low at ~6 pg/ml.^17^ These estrogens are primarily synthesized by peripheral conversion of androstenedione (0.53 ng/ml) and testosterone (22.2 ng/dl), the androgens produced from postmenopausal ovaries and adrenal glands.^25^ Postmenopausal androgen biosynthesis, albeit slightly reduced after menopause, remains comparable to premenopausal levels of androstenedione (0.83 ng/ml) and testosterone (25.8 ng/dl).^15^ Obesity is also associated with elevated levels of estrogens and increased breast cancer risk in postmenopausal women.^26^ In premenopausal women, however, obesity is linked to a reduced risk of breast cancer with lower levels of progesterone.^27^ It remains unclear whether progesterone levels are elevated in postmenopausal women with obesity as are estrogen levels.^26^ Owing to the limited sensitivity of progesterone assay, it has been challenging to measure accurate concentrations of progesterone in postmenopausal women.^23^ Studies indicate that the ovary and adrenal gland are likely to be primary sources of postmenopausal progesterone.^28,29^

## HISTORICAL PERSPECTIVE ON ESTROGENS AND BREAST CANCER

Even before the discovery of estrogen, ovarian hormones were speculated to be involved in breast cancer. In 1896, the British surgeon George Beatson observed a regression of metastatic breast cancer in premenopausal women after oophorectomies.^30^ Together with animal studies linking estrogen to mammary tumor development, estrogen was postulated to increase breast cancer risk in humans.^31^

E_1_ was the first estrogen discovered through purification from pregnant women’s urine in 1929.^32^ In the 1930s and early 1940s, other natural estrogens, including conjugated equine estrogens (CEEs), were extracted from the urine of pregnant women or pregnant mares.^33^ Estrogens were also synthesized—notably, diethylstilbestrol (DES) and ethinylestradiol (EE).^33^ These natural and synthetic estrogens were used for menopausal symptoms and gained broad popularity in the late 1960s.^33,34^ There was a brief decrease in use between 1975 and 1980 due to reports of increased endometrial cancer risk associated with estrogen-only formulations.^35–37^ With the addition of a progestin to counteract the risk of endometrial cancer,^38^ HRT use had continued to rise through the 1980s and 1990s, coupled with the Food and Drug Administration (FDA) approval for osteoporosis prevention in 1988.^34,39,40^ Following a peak in the late 1990s, HRT use had sharply declined worldwide in the aftermath of the reports of the Women’s Health Initiative (WHI) trials in 2002 and 2004, which, at that time, indicated that excess health risks of HRT would outweigh its benefits.^3,41–43^ Despite a series of follow-up WHI studies reversing the health concerns of HRT,^4,44,45^ HRT use has remained controversial, primarily owing to the concern of breast cancer.^11^

It was a serendipitous contamination that introduced estrogen into oral contraceptives during the birth control pill trials in the 1950s.^46^ The ‘birth control pill’ was originally based on the observations that high levels of exogenous progesterone would mimic pregnancy and thereby block ovulation and conception.^46^ Though effective in preventing pregnancy, the ‘pill’ composed of a purified progestin alone also caused bleeding in the uterus, which resulted from irregular shedding of the endometrium.^46,47^ Fortuitously, it was discovered that norethynodrel, one of progestin compounds of interest at that time, did not cause uterine breakthrough bleeding and was later found to be contaminated with mestranol, a synthetic estrogen.^46^ This observation paved the way for adding an estrogen to maintain endometrial stability and thus reduce the risk of uterine breakthrough bleeding.^48^ Moreover, it was later recognized that estrogen contributed to the contraceptive effects of the pill.^1,47,48^

Early formulations of high-dose contraceptive estrogens caused multiple undesirable side effects, including venous thromboembolism,^1^ prompting a dose reduction in estrogens. Estrogen doses were reduced from mestranol 150 μg in an original formulation to estrogen <50 μg by the early 1980s.^49^ Most modern oral contraceptives now typically contain 20–35 μg EE.^50^ Progestins have also changed over the years to newer formulations to minimize androgenic side effects.^51^ The original formulation of norethynodrel 10 mg was reduced in subsequent generations of progestins, such as levonorgestrel (LNG) 100–250 μg and desogestrel 150 μg.^48^ Following the FDA approval in 1960 of the first combination pill (norethynodrel 5 mg and mestranol 75 μg), a progestin-only pill was also introduced in 1973 to avoid the adverse effects of estrogen.^49^ A progestin-only pill typically contains lower progestin doses (‘minipill’) than progestin–estrogen combination products.^52^

Unlike its secondary role in oral contraceptives, estrogen was the main component of HRT, aimed to counteract for the sharp decline of menstrual estrogens after menopause.^4^ While relieving menopausal symptoms, the stimulatory effect of estrogen-alone HRT on endometrial proliferation and cancer risk required the addition of a progestin in postmenopausal women with an intact uterus.^3,53,54^

Once hailed as the ‘fountain of youth’, exogenous estrogens began to raise concerns for breast cancer risk.^34^ To address these concerns, large national and international studies—notably, the Nurses’ Health Study in 1976, the Collaborative Group on Hormonal Factors in Breast Cancer (CGHFBC) in 1992, and the WHI in 1992—were launched to examine the impacts of hormonal contraceptives and HRT on breast cancer risk. Overall, these studies reported a small increase in breast cancer risk among users of hormonal contraceptives.^55–58^ Initial reports of HRT studies generally indicated that estrogen therapy, particularly when given in combination with a progestin, was associated with a moderately increased risk of breast cancer in postmenopausal women.^3,40,44,59–62^

## ESTROGEN AND BREAST CANCER—INTRICATE COMPLEXITY

Multiple lines of evidence have suggested a link between estrogen and breast cancer.^8,9^ Inhibiting estrogen signaling with the selective estrogen receptor (ER) modulator tamoxifen, degrading the ER by fulvestrant, and blocking estrogen biosynthesis by aromatase inhibitors reduce the risk of recurrence or progression in patients with ER-positive breast cancer.^63–68^ Blocking ER signaling by antiestrogens, such as tamoxifen and aromatase inhibitors, was also shown to reduce breast cancer risk.^69–74^ Additionally, high blood levels of estrogens have been linked to an increased risk of breast cancer.^8,75^ Breast epithelial cell proliferation is increased in postmenopausal women taking estrogen-containing HRT,^76^ as well as in mice and primates treated with estrogen,^77–80^ supporting estrogen as an oncogenic factor for breast cancer.^8,81^ Current evidence thus appears to support the encompassing oncogenic and tumor-promoting role of estrogen in breast cancer risk, recurrence, and growth/progression.

Yet a more careful look reveals a complex, counterintuitive relationship between estrogens and breast cancer. Estrogens, such as DES and E_2_ given at high doses (1.5–1500 mg/day), had been used to treat ER-positive advanced breast cancer until the introduction of tamoxifen,^82–86^ an ER antagonist in breast tissue. After a failed development as a contraceptive, tamoxifen was reborn as a therapy for estrogen-driven breast cancer with an FDA approval in 1977.^63,87^ The efficacy of DES therapy in postmenopausal women with metastatic breast cancer was comparable to or higher than that of tamoxifen (response rates, 41% versus 33%),^84^ yet tamoxifen was more tolerable.^83–85^ E_2_ treatment results in growth arrest and apoptosis in estrogen-deprived cultured ER-positive breast cancer cells and may thus explain its therapeutic effect.^86,88^

Similarly, high doses of progestins, such as medroxyprogesterone acetate (MPA, 500–1500 mg/day) and megestrol acetate (60–180 mg/day), showed response rates (28%) analogous to tamoxifen (31%, 20 mg/day) in postmenopausal women with ER-positive metastatic breast cancer.^89–92^ This may be in part through partial agonistic activity for the glucocorticoid receptor^93,94^ and the androgen receptor.^81,95^

Endogenous estrogens—E_2_ and E_1_—are hydroxylated and further conjugated to form various metabolites, including 2-hydroxyestrone, 2-hydroxyestradiol, 4-hydroxyestrone, 16α-hydroxyestrone, 2-methoxyestrone, and quinones.^96,97^ Epidemiologic studies have consistently shown that endogenous estrogens or collective estrogen metabolites are associated with an increased risk of breast cancer among postmenopausal women.^8,97–101^ In contrast, the association between individual estrogen metabolites and breast cancer risk appears to be inconsistent.^97,100,101^ It is possible that individual estrogen metabolites may function as oncogenic or onco-protective in breast cancer.^96^ The causal connection between estrogen metabolites and breast cancer risk remains to be established.

## BREAST CANCER RISK FROM CURRENT HORMONAL CONTRACEPTIVES IN PREMENOPAUSAL WOMEN

Overall, hormonal contraceptives are associated with a small increase in breast cancer risk in young women under age 50 years^55–58,102–104^ (Table 1). A nationwide study of all women (1.8 million) aged 15–45 years in Denmark reported a 20% relative increase in breast cancer risk among current and recent users of hormonal contraception compared with nonusers [relative risk (RR) 1.20, 95% confidence interval (CI) 1.14–1.26, *P* = 0.002].^105^ A 20% increase in the RR of breast cancer should be taken into perspective because absolute breast cancer risks are quite low among young women who use hormonal contraceptives. In the general population, the risks of developing invasive breast cancer over the subsequent 10 years for women ages 20, 30, and 40 years are estimated to be 0.07%, 0.5%, and 1.6%, respectively.^106^ A 20% increase with hormonal contraception in these age groups would therefore elevate their respective 10-year breast cancer risks to 0.08%, 0.6%, and 1.9%.

Importantly, it appears that breast cancer risk from oral contraceptive use may be primarily attributed to progestins. Large clinical trials, comparing breast cancer risks between progestin–estrogen combined contraceptives and progestin-only contraceptives, indicate that estrogens in hormonal contraceptives may have little or no impact on breast cancer risk.^105,107^ A Swedish study of 1.5 million women aged 15–34 years reported a significant increase in breast cancer risk with progestin-only contraception [incident rate ratio (IRR) 1.32, 95% CI 1.20–1.45], which was not observed with combined hormonal contraceptives (IRR 1.03, 95% CI 0.91–1.16).^108^ Notably, the progestin LNG exhibited a higher risk of breast cancer in progestin-only pills (RR 1.93, 95% CI 1.18–3.16) than did in LNG–estrogen combined pills (RR 1.33, 95% CI 1.20–1.48) (nationwide Danish study) (Table 1),^105^ despite the fact that LNG doses range from 100 to 150 μg for combined formulations and 30 μg in progestin-only pills.^48,52^

Some studies and a meta-analysis have shown a 20%-30% increase of breast cancer risk associated with use of progestin-releasing intrauterine devices (IUDs), which release a constant low dose of progestin (Table 1).^105,107^ In contrast, such an increase was not found in a meta-analysis of 190 475 women who used LNG-IUDs.^109^ The contraceptive effects of progestin-only pills (30–350 μg/day) and progestin-releasing IUDs (6–20 μg LNG release/day) are primarily achieved by acting directly on the cervix to increase the viscosity of cervical mucus, preventing sperm migration.^110–112^ Yet some of these progestin-only contraceptives were reported to inhibit ovulation in up to 40%-60% of users,^113–115^ indicating a potential systemic effect. A recent large study of LNG-IUDs showed an HR of 1.4 (95 CI 1.2–1.5) for breast cancer risk in 78,595 LNG-IUD users, compared with the matched 78,595 nonuser control group.^116^ Numerically, this increased risk resulted in an excess of 14 breast cancer diagnoses per 10,000 users. Larger prospective studies may be needed to truly determine the effect of IUDs and their extent on the risk of breast cancer.

Use of combined oral contraceptive pills is associated with markedly reduced risks (30%-50%) of ovarian and endometrial cancers.^6,117–120^ Thus, juxtaposed with the efficacy and safety for contraception as well as the significant risk reduction in ovarian and endometrial cancers, the breast cancer risk posed by hormonal contraceptives is small and does not appear to increase mortality in premenopausal women.^67^

## ESTROGEN-CONTAINING HRT AND BREAST CANCER RISK IN POSTMENOPAUSAL WOMEN

HRT contains much higher doses of an estrogen (e.g. CEEs, 0.625 mg/day) and a progestin (e.g. MPA, 2.5 mg/day) than those in hormonal contraceptives.^3^ The progestin component was added to reduce the risk of endometrial hyperplasia in women with an intact uterus.^3,53,54^ Hence, estrogen-alone HRT is generally used in women with prior hysterectomy.

Overall, as summarized in Table 2, initial observational studies of HRT showed that estrogen-alone therapy was associated with smaller increases (6%-37%) in breast cancer risk than was estrogen-plus-progestin therapy (17%-131%) in postmenopausal women (aged ≥50 years) compared with never-users.^40,59–61^ In contrast with the observational studies, several placebo-controlled RCTs, including the WHI trial, have shown that estrogen-alone HRT has little or no impact on elevating breast cancer risk in postmenopausal women.^44,121–123^ Notably, with longer than 20 years of follow-up, the WHI study showed that estrogen-alone HRT had significantly and durably reduced breast cancer risk by 22% (range 21%-69%) [hazard ratio (HR) 0.78, 95% CI 0.65–0.93, *P* = 0.005] as well as breast cancer mortality by 40% (HR 0.60, 95% CI 0.37–0.97, *P* = 0.04) in postmenopausal women with hysterectomy.^43,44,124^ Consistent with the observational studies, however, the WHI RCT affirmed that estrogen plus progestin increased the risk for breast cancer by 28% (range 22%-36%) (HR 1.28, 95% CI 1.13–1.45, *P* < 0.001).^3,44^

The inconsistency or discrepancy in the breast cancer risks of estrogen-alone HRT found between observational studies and RCTs may be due to a difference in baseline breast cancer risks between exposure and control groups in observational studies.^125^ In RCTs, randomization would make baseline breast cancer risks similar between estrogen-alone HRT and placebo groups, whereas the absence of randomization may make observational studies subject to potentially different baseline risks for breast cancer between users of estrogen-alone HRT and never-users (control group).^125^ If true breast cancer risk from estrogen-alone HRT were small or absent, the baseline risk difference in observational studies could have a sizable impact on the risk difference measured in RCTs.^125,126^ From observational studies to WHI RCTs, there were overall decreases in breast cancer risks for estrogen-alone HRT (6%-37% increase to 22% decrease) and estrogen-plus-progestin HRT (17%-131% increase to 22%-36% increase), suggesting a baseline risk difference between HRT users and never-users in non-randomized, observational studies. When this baseline risk difference in breast cancer was removed or mitigated by randomization, the weak association of estrogen-alone HRT with increased breast cancer risk did not appear to remain.^43,44,124^ Even after randomization, however, estrogen-plus-progestin HRT, which exhibited a robust association with increased breast cancer risk in observational studies, still showed a significant increase in breast cancer risk.^3,44^

Collectively, these findings support a causative role of progestins from HRT, with minimal effect from estrogen, in elevating breast cancer risk.

## IMPACT OF ENDOGENOUS ESTROGENS AND PROGESTERONE ON HRT-ASSOCIATED BREAST CANCER RISK

Bilateral oophorectomy (BO) has been shown to decrease breast cancer risk.^127,128^ Surgical or chemical deprivation of ovarian hormones has been associated with a decrease in breast cancer risk, recurrence, and death.^129–133^ Also, risk-reducing oophorectomy in *BRCA1/2* carriers leads to a significant reduction (by 40%-50%) of breast cancer risk and death in most studies.^134^ These findings suggest a causal role of endogenous ovarian hormones in breast cancer risk.

In the WHI trial of postmenopausal women with hysterectomy, estrogen-alone HRT showed no increase in breast cancer risk (HR 0.84, 95% CI 0.54–1.33) compared with placebo in women with BO after 18 years of follow-up.^135^ In the Nurses’ Health Study, women with both oophorectomy and hysterectomy showed a decreased risk of breast cancer (HR 0.75, 95% CI 0.68–0.84) compared with women with hysterectomy alone.^128^ In this study, estrogen-alone HRT was more prevalently used in women with oophorectomy (78.3%) than in women without oophorectomy (36.0%).^128^ The Nurses’ Health Study also reported that estrogen-alone HRT was not associated with increased breast cancer risk in women with BO.^136^ Together, these findings suggest that estrogen-alone HRT is not likely to increase breast cancer risk in the absence of ovarian hormones.

Deprivation of ovarian hormones appears to eliminate or neutralize any potential breast cancer risk from estrogen-alone HRT. *BRCA1* carriers who underwent BO before age 45 years and received estrogen-alone HRT showed a nonsignificant yet numerical reduction of breast cancer risk (HR 0.47, 95% CI 0.20–1.15, *P* = 0.1), vis-à-vis their counterparts not taking HRT^137^ (Table 2). In contrast, estrogen-plus-progestin or progestin-alone HRT following oophorectomy was associated with a significant increase in breast cancer risk in *BRCA1* carriers (HR 3.38, 95% CI 1.17–9.73, *P* = 0.02).^137^

Depletion of ovarian hormones also offers insights into the role of endogenous estrogens and progesterone in breast cancer risk associated with HRT. In postmenopausal women, oophorectomy further reduces estrogen levels (by 40% for E_1_; by 37% for E_2_) similar to concentrations in premenopausal women following oophorectomy.^15^ The observations that estrogen-alone HRT has little to no impact on breast cancer risk after depletion of ovarian hormones suggest that endogenous estrogens alone are not likely to elevate breast cancer risk. The increased risk of breast cancer by estrogen-plus-progestin HRT after depleting ovarian hormones points to endogenous progesterone as an oncogenic hormonal driver for breast cancer. As estrogen is known to induce progesterone receptor (PgR) expression in mammary epithelial cells,^138–140^ exogenous and endogenous estrogens may also contribute to breast cancer risk by augmenting progesterone signaling.^141^ Endogenous testosterone, whose levels are significantly reduced post-oophorectomy,^15^ is unlikely to account for the progestin-mediated increased breast cancer risk.

## NO INCREASED RISK OF BREAST CANCER BY ESCALATED LEVELS OF ESTROGENS FROM OVARIAN STIMULATION

Despite a short duration, fertility treatments, such as IVF or assisted reproductive technology (ART), have raised a concern for a potential increase in breast cancer risk, because such treatments markedly increase the endogenous production of estrogens.^142,143^ IVF or ART commonly involves ovarian stimulation by the administration of gonadotropins to render the maximum number of eggs harvested in a single IVF cycle.^144^ During an IVF cycle, gonadotropins spur the growth and maturation of multiple follicles, simultaneously, into preovulatory follicles, thereby releasing supraphysiological levels of particularly E_2_ (500-4000 pg/ml) in the follicular phase.^145–147^ After the final maturation of the oocytes in the preovulatory follicles by an injection of human chorionic gonadotropin, the oocytes are recovered via follicle aspiration.^146^ The luteal phase of an IVF cycle, however, is defective due to inadequate corpus luteum function leading to insufficient progesterone production.^148^ Typically, the luteal-phase defect requires the administration of exogenous progesterone to prepare the endometrium for the implantation of transplanted embryos and to improve pregnancy rates.^148^ Thus, women undergoing an IVF or ART procedure are exposed to transiently yet acutely elevated concentrations of endogenous estrogens, as high as 10 times the levels in a normal menstrual cycle.^145,146^

Recent meta-analyses, which examined the impact of ovarian stimulation and hormone fertility treatment on breast cancer risk, found no significantly increased risk of breast cancer (odds ratio 0.97, 95% CI 0.90–1.04).^142,143^ A large cohort study, assessing 255 786 women in the UK treated with ART, indicated that the absence of breast cancer risk increase in women with IVF/ART was not attributed to pregnancy/live births.^149^ Additionally, in a Dutch population-based study, breast cancer risk was significantly reduced with 7 or more cycles of IVF or fertility treatments compared with 1–2 cycles (HR 0.55, 95% CI 0.39–0.77).^150^ Moreover, ovarian stimulation was associated with significantly reduced risks of breast cancer recurrence (RR 0.58, 95% CI 0.46–0.73) and mortality (RR 0.54, 95% CI 0.38–0.76) in young women diagnosed with breast cancer who sought oocyte/embryo cryopreservation, and a reduced risk of recurrence (RR 0.34, 95% CI 0.17–0.70) in breast cancer survivors receiving ART.^151^ While the mechanism is unclear for these seemingly counterintuitive breast cancer risk reductions and survival benefits, collective evidence suggests that high levels of estrogens induced by ovarian stimulation appear to have no adverse effect on breast cancer risk or recurrence.

## REPRODUCTIVE FACTORS AND BREAST CANCER RISK

The oncogenic roles of endogenous hormones are indicated by the association of reproductive factors—such as pregnancy, lactation, menarche, and menopause—with breast cancer risk.^152–154^

Parous women are generally at a lower risk of breast cancer than nulliparous women.^155,156^ This protection of pregnancy against breast cancer is largely attributable to an early age at first pregnancy.^155,156^ While pregnancy at younger ages (20–30 years) shows a protective effect against breast cancer risk, this protection may be lost in later-age pregnancies.^155,156^ First full-term pregnancy at an age older than 30–35 years is associated with an increased risk of breast cancer.^155,156^

The mechanism underlying this pregnancy-associated breast cancer risk reduction appears to be complex.^157,158^ Observational data suggest that the timing of pregnancy (i.e. early age at first pregnancy), rather than parity, is the most important determinant of the breast cancer risk reduction linked to pregnancy.^155,156^ Thus, the pregnancy-associated protective effect might be hormone independent. Considering the treatment effects of high-dose estrogen and progestin therapies in advanced breast cancer,^86,92^ it may also be possible that high physiological levels of pregnancy estrogens and progesterone might act as anti-carcinogenic in breast tissue. Other putative mechanisms include pregnancy-driven alterations in breast cells, spurring a switch from an undifferentiated to a more differentiated state resistant to carcinogenesis.^157,158^

Most studies have indicated that pregnancy does not affect the risk of recurrence or death among breast cancer survivors.^159,160^ Breast cancer survivors with pregnancy had significantly better overall survival than those without pregnancy (HR 0.46, 95% CI 0.27–0.77, *P* < 0.05).^160^ Thus, pregnancy appears to be not only safe for breast cancer survivors, but also may potentially be protective against breast cancer recurrence. These data suggest that pregnancy-associated increases in steroid hormone synthesis may not have adverse effect on breast cancer recurrence.

An early onset of menarche and a late onset of menopause are associated with an increased risk of breast cancer,^161^ while lactation or breastfeeding is associated with a decreased risk of breast cancer.^162^ Early-age menarche and late-age menopause would increase exposure to menstrual hormones. As lactation inhibits the menstrual cycle and ovulation, it would reduce menstrual hormone exposure.^162^ Collectively, these findings support an oncogenic role of menstrual hormones in breast cancer.

## ONCOGENIC ROLE OF PROGESTERONE IN BREAST CANCER

Estrogen is the major mitogenic hormone in pubertal murine mammary glands.^77,163^ In adult mice, however, estrogen has little impact on mammary epithelial proliferation while eliciting a notable induction of PgR expression in mammary epithelial cells.^138–140^ In contrast, progesterone, particularly when treated with E_2_, robustly stimulates adult mammary epithelial proliferation in ovariectomized mice and primates.^77–79,140,164^ Normal breast epithelial cells significantly increase proliferation by 62%-103% in the luteal phase compared with the follicular phase.^165^ Additionally, progesterone levels are significantly and positively correlated with breast epithelial cell proliferation.^165^ Collectively, animal and human studies indicate that progesterone is the principal hormonal factor driving adult mammary/breast epithelial proliferation.^76,141^

The potent mitogenic role of progesterone in the mammary epithelium is consistent with the oncogenic impact of progestins in elevating breast cancer risk in clinical studies of HRT and hormonal contraceptives.^40,44,59–61,105,107^ In particular, the Nurses’ Health Study showed a significantly elevated risk of breast cancer among postmenopausal women using progestin-alone HRT (RR 2.24, 95% CI 1.26–3.98) compared with never-users.^166^ Employing a sensitive progesterone assay, a recent study reported an increased risk of breast cancer in postmenopausal women with higher blood levels of progesterone compared with lower progesterone levels (HR 1.24, 95% CI 1.07–1.43, *P* = 0.004).^23^ The effects of antiprogestins to prevent mouse mammary tumors, as well as ovarian cancer in mice, lend further support to the oncogenic role of endogenous progesterone and progestins in breast cancer.^167,168^

It is worth noting that bioidentical progesterone may confer a lower risk of breast cancer than progestins.^169^ Micronized progesterone, used along with estrogen HRT, was not significantly associated with an increased risk of breast cancer when used up to 5 years (HR 1.13, 95% CI 0.99–1.29).^170^ Breast cancer risk, however, was significantly elevated with long-time use of estrogen plus micronized progesterone (>5 years) (HR 1.31, 95% CI 1.15–1.48), albeit less than with estrogen–progestin HRT (HR 2.02, 95% CI 1.81–2.26).^170,171^ Accordingly, the progestin MPA exhibits higher proliferative activity in mammary epithelial cells than does progesterone.^78^

## MECHANISM UNDERLYING THE PREVENTIVE AND THERAPEUTIC EFFECTS OF ANTIESTROGENS

In large randomized preventive trials of tamoxifen and the aromatase inhibitor anastrozole, these endocrine therapies have reduced the risk of ER-positive breast cancer by 30%-50%, particularly in women with an increased risk of breast cancer.^69–74^ However, tamoxifen did not reduce breast cancer risk in women who never used estrogen-alone HRT (RR 1.00, 95% CI 0.67–1.50).^71^ Counterintuitively, the tamoxifen-mediated risk reduction of breast cancer was pronounced among women who also used estrogen-alone HRT (RR 0.43, 95% CI 0.2–0.95).^71^

Tamoxifen has dual properties as antiestrogenic and estrogenic in breast tissue and breast cancer, depending upon its concentrations and biological context.^164,172,173^ Absent estrogen, tamoxifen exhibits partial estrogenic activity,^164^ whereas in the presence of estrogen, tamoxifen acts as an antiestrogen that decreases estrogen-induced PgR expression.^174–176^ Accordingly, tamoxifen exhibited higher sensitivity to ER-positive/PgR-positive breast tumors than ER-positive/PgR-negative breast tumors.^177^ Similarly, endocrine therapy was more effective in reducing recurrence and mortality for breast tumors expressing both ER and PgR (ER positive/PgR positive) than ER-positive/PgR-negative breast tumors.^177–179^ Fittingly, tamoxifen resistance was associated with a loss of PgR expression in ER-positive/PgR-positive breast tumors.^180^

Together, these findings suggest that the risk-reducing and therapeutic effects of antiestrogens may be mediated by the ability of antiestrogens to suppress progesterone signaling.

## HORMONAL FACTORS FOR BREAST CANCER RECURRENCE AND PROGRESSION

The therapeutic effects of antiestrogens, such as tamoxifen and aromatase inhibitors, on reducing breast cancer recurrence and progression have naturally pointed to estrogen as a main hormonal driver for the development, growth, and progression of ER-positive breast cancer.^66,181^ This concept dates back to the discovery in the late 19th century linking oophorectomies to regression of metastatic breast cancer in premenopausal patients.^30,182^ This observation led to a seeming logical conclusion that the therapeutic effect of oophorectomy for patients with metastatic breast cancer was derived from the deprivation of ovarian estrogens while presuming an absence of benefit for ER-negative breast tumors.^183–185^

At odds with this notion, further data showed therapeutic effects of oophorectomy in both ER-positive (response rate, 71%) and ER-negative (21%) metastatic breast cancers.^186^ Similarly, oophorectomy was associated with significant reductions (55%-62%) in breast cancer mortality among *BRCA1/2* carriers with nonmetastatic ER-positive or ER-negative breast cancer.^131,187^ Notably, the protective effect of oophorectomy against breast cancer death was even more pronounced in ER-negative breast cancer (HR 0.07, 95% CI 0.01–0.51, *P* = 0.009) than in ER-positive breast cancer (HR 0.48, 95% CI 0.25–0.86, *P* = 0.01).^131,187^ Collectively, these findings suggest that oophorectomy (i.e. deprivation of ovarian hormones) reduces the recurrence of ER-positive as well as ER-negative breast cancers.

Plausible alternative explanations may be the culpable effects of ovarian progesterone. Its deprivation complemented by the loss of ovarian estrogens may be the primary reason for the recurrence suppression and the reduced breast cancer mortality post-oophorectomy in *BRCA1/2* carriers with ER-positive or ER-negative nonmetastatic breast cancer. Similarly, depletion of both ovarian progesterone and estrogens would likely be responsible for the tumor regression observed in ER-positive and ER-negative metastatic breast cancers after therapeutic oophorectomy.^186^ Also, the reason for tumor regression in only 20%-50% of patients with metastatic breast cancer following oophorectomy^183,186,188^ may be attributable to non-hormonal causes of tumor progression rather than the rest being ER-negative tumors.

## HRT FOR BREAST CANCER SURVIVORS

Further corroborating evidence on the oncogenic role of progestogens in breast cancer may be gleaned from two independent RCTs that assessed the breast cancer risk of HRT among breast cancer survivors in Sweden: the HABITS and the Stockholm trials.^189,190^ The participants of these trials were postmenopausal women without evidence of recurrence after the diagnosis and treatment of non-metastatic breast cancer. After HRT versus no treatment of 2–5 years and median follow-ups of 2–10 years, the HABITS trial showed a significant increase of recurrence (HR 2.4, 95% CI 1.3–4.2),^189,191^ whereas the Stockholm trial reported no significant impact of HRT on breast cancer recurrence (relative hazard 0.82, 95% CI 0.35–1.9).^190,192^ No mortality difference was observed in both trials.

There were two major differences between these trials: progestin exposure and tamoxifen use. In the HABITS trial, nearly half (46%) of the participants were treated with continuous estrogen–progestin HRT with 47% for estrogen alone or combined with low exposure of a progestin.^189^ In contrast, as the Stockholm trial was specifically designed to minimize progestin exposure from HRT, 95% of the participants were treated with estrogen alone or combined with low exposure of a progestin.^190^ Thus, investigators of these trials attributed the increased risk of recurrence in the HABITS trial to higher exposure of progestins.^192^ Also, more women were treated with tamoxifen as an adjuvant therapy in the Stockholm trial (52%) than in the HABITS trial (21%). The risk-reducing effect of breast cancer by tamoxifen was promoted by estrogen-alone HRT,^71^ which would induce PgR and thus potentiate the anti-breast-cancer effect of tamoxifen via suppression of progesterone signaling.^174–176^ Thus, higher use of tamoxifen, combined with progestin-limited estrogen HRT, may have also mitigated the risk of breast cancer recurrence in the Stockholm trial.

A recent combined analysis of RCTs, prospective, and retrospective studies showed no increase in breast cancer recurrence (RR 0.85, 95% CI 0.54–1.33) and mortality (RR 0.91, 95% CI 0.38–2.19),^193^ albeit with disagreements among studies.^194–197^ While HRT is generally contra-indicated for breast cancer survivors,^198^ collective evidence cautiously suggests that estrogen therapy, combined with minimal use of a progestogen, might be a safe option for these women in need of HRT.

## CONCLUSIONS

There is a long-held notion that estrogen exposure will increase the risk for breast cancer. Conversely, a body of emerging clinical and basic research evidence suggests that progestogens (synthetic or endogenous progesterone) are most likely the primary hormonal factor underlying seemingly estrogen-associated breast cancer risk. Progestogens appear to be the principal hormone driving the development and recurrence of breast cancer, while estrogens may contribute to breast cancer risk by amplifying progesterone signaling.

It has been well recognized that HRT provides an array of health benefits—including alleviation of menopausal symptoms, mood improvement, weight control, and prevention of bone fractures.^4,5,199,200^ There are, however, risks that need to be weighed for HRT use. Estrogen-alone HRT may increase endometrial cancer risk in postmenopausal women with an intact uterus, while estrogen-plus-progestin therapy could increase breast cancer risk in these women. Therefore, the risks and benefits of HRT should be carefully assessed for individual women.^201^ If a woman with an average risk for breast cancer and an intact uterus, for instance, experiences significant menopausal symptoms, she may opt for estrogen, combined with minimal use of micronized progesterone or progestin–IUD to counter potential endometrial hyperplasia. If she is more concerned about breast cancer risk than endometrial cancer risk, she may choose estrogen alone at a lower dose and regularly monitor endometrial growth. In postmenopausal women seeking HRT after a hysterectomy alone or in combination with an oophorectomy, estrogen alone would relieve menopausal symptoms with minimal cancer risks.

Overall, current scientific evidence suggests that estrogen therapy, coupled with minimal use of a progestogen, is likely to offer net health benefits in women in need of HRT.

## Figures and Tables

**Table 1.** Hormonal contraceptives and breast cancer risk

	CGHFBC		US Nurses’ Health II		Denmark		UK CPRD		*BRCA1/2* carriers	
Study type	Meta-analysis of 54 epidemiological studies		Prospective		Nationwide prospective cohort		Nested case-control study and meta-analysis (12 studies)		Review (10 BC studies)	
HC type	COCP (P + E) (formulations from 1960s to 1980s)		COCP/POP distinction not provided; likely most use from COCP		COCP (P + E); P only (POP, I-P, P-Imp, P-IUD); COCP, ~90% of HC use		P only (POP, I-P, P-Imp, P-IUD); COCP (P + E)		COCP (P + E)	
HC formulation	E (EE, < or ≥50 μg/day; M, ≥50 μg/day); P (LNG, < or >250 mg/day; NET, < or >1000 mg/day)		Formulations from 1980s and 1990s		Formulations since 1995; E (EE, 20–50 μg/day); P (CPT, DRSP, DSG, GSD, LNG, NET, NGM), dose varied		Formulations since 1990s; P (CPT, DRSP, DSG, GSD, LNG, NET, NGM, NGT), dose varied		BC risk	
HC duration, years	<1 to ≥15; Median, 3		<1 to ≥8		<1 to >10		<1 to ≥5		*BRCA1*	*BRCA2*
Participants	153 536 (total): 53 297 (BC cases), 100 239 (controls)		116 608 (total): 1344 (BC cases), 115 264 (controls)		1 797 932 (total): 11 517 (BC cases), 1 786 415 (controls);		27 669 (total); 9498 [BC cases: 4195 from HC (53% P only)]; 18 171 [controls: 7092 from HC (52% P only)]		1.09 (0.77–1.54) RR (95% CI)	1.15 (0.61–2.18) RR (95% CI)
Mean age (SD), years	Median age and year at BC diagnosis, 49, 1984; Median age for HC use, 26 (range: early teens to early 40s)		Age range at enrollment (1989): 25–42		Age range at BC diagnosis: 15–49		Mean age at BC diagnosis (1996–2017), 43 (5); Age range at BC diagnosis, 20–49		**1.49 (1.05–2.11) HR (95% CI)**	**2.58 (1.21–5.49) HR (95% CI)**
Observation period, years	Original studies published from 1980 to1995		~ 12 (1989–2001)		Mean, 10.9 (SD 5.8); 1995–2012		Before BC diagnosis; mean, 7.3 (SD 4.6)		1.08 (0.94–1.25) OR (95% CI)	1.03 (0.81–1.32) OR (95% CI)
BC risk^a^ overall	**1.24 (1.15–1.33) RR (95% CI) current use**	1.07 (SD 0.02) ever use	**1.33 (1.03–1.73) RR (95% CI)** current use	1.12 (0.95–1.33) RR (95% CI) past use	**1.20 (1.14–1.26) RR (95% CI)**		**1.25 (1.18–1.33) OR (95% CI) Case ctrl**	Meta-analysis RR (95% CI)	1.19 (0.92–1.55) OR (95% CI)	1.36 (0.89–2.10) OR (95% CI)
BC risk by age/HC type/HC duration	1.18 (SD 0.122) ≤1 year current use	**1.16 (1.08–1.23) 1–4 years after last use**	1.16 (0.80–1.69) >0–8 years current use	**1.42 (1.05–1.94) ≥8 years** current use	**1.21 (1.11–1.33) LNG-IUD** ^b^	**1.08 (1.03–1.13)** Past HC use > 6 months	**1.23 (1.14–1.32) COCP**	N/A	**1.45 (1.20–1.75) OR (95% CI)**	—
	1.27 (SD 0.079) 1–4 years current use	**1.07 (1.02–1.13) 5–9 years after last use**	0.81 (0.45–1.45) NET (P) current use	0.50 (0.18–1.35) T-NET (P) current use	1.09 (0.80–1.50) NET (P) COCP	1.00 (0.80–1.25) NET (P) POP	**1.26 (1.16–1.37) POP**	**1.29 (1.21–1.37) POP**	0.78 (0.59–1.04) ES (95% CI)	1.04 (0.81–1.32) ES (95% CI)
	1.21 (SD 0.061) 5–9 years current use	1.01 (0.96–1.05) ≥10 years after last use	0.86 (0.32–2.34) LNG (P) current use	**3.05 (2.00–4.66) T-LNG (P)** current use	**1.33 (1.20–1.48) LNG (P) COCP**	**1.93 (1.18–3.16) LNG (P) POP**	**1.25 (1.07–1.45) I-P**	**1.18 (1.07–1.30) I-P**	1.24 (0.45–3.40) HR (95% CI)	0.71 (0.21–2.37) HR (95% CI)
	1.29 (SD 0.060) ≥10 years current use		1.34 (0.79–2.28) NETA (P) current use	1.22 (0.45–3.32) ED (P) current use	**1.12 (1.01–1.25) DSG (P) COCP**	1.18 (0.87–1.60) DSG (P) POP	1.22 (0.93–1.59) P-Imp	**1.28 (1.08–1.51) P-Imp**	1.08 (0.75–1.5) HR (95% CI)	**1.75 (1.03–2.9) HR (95% CI)**
	1.30 (SD 0.089) current use nulliparous	1.23 (SD 0.042) current use parous	**1.89 (1.05–3.41) NGT (P)** current use		1.01–1.62 (RR range by P type) COCP	1.00–1.93 (RR range by P type) POP	**1.32 (1.17–1.49) LNG-IUD**	**1.21 (1.14–1.28) LNG-IUD**	**1.7 (1.1–2.05) HR (95% CI)**	
References	*Lancet and Contraception*. 1996^102,103^		*Cancer Epidemiol Biomarkers Prev*. 2010^57^		*N Engl J Med*. 2017^105^		*PLoS Med*. 2023^107^		*Arch Gynecol Obstet*. 2020^104^	

Progestin-only contraceptives are highlighted in light orange. Statistically significant risk is represented in boldface.

BC, breast cancer; Case ctrl, case-control study; CGHFBC, Collaborative Group on Hormonal Factors in Breast Cancer; CI, confidence interval; COCP, combined oral contraceptive pill; CPRD, Clinical Practice Research Datalink; CPT, cyproterone; DSG, desogestrel; DRSP, drospirenone; E, estrogen; ED, ethynodiol diacetate; EE, ethinylestradiol; ES, effect estimate; GSD, gestodene; HC, hormonal contraceptive(s); HR, hazard ratio; I-P, injected progestin; LNG, levonorgestrel; M, mestranol; N/A, information not available; NET, norethisterone; NETA, norethisterone acetate; NGT, norgestrel; NGM, norgestimate; OR, odds ratio; P, progestin; P-Imp, progestin implant; LNG-IUD, progestin LNG-releasing intrauterine device; POP, P-only pill; RR, relative risk; SD, standard deviation; T-LNG, triphasic LNG; T-NET, triphasic NET.

a BC risk estimated using cases of invasive breast cancer.

b An updated analysis of LNG-IUD showed an HR of 1.4 (95 CI 1.2–1.5) for breast cancer risk in 78,595 LNG-IUD users regardless of duration of use, compared with the matched nonuser control group. Numerically, this resulted in an excess of 14 breast cancer diagnoses per 10,000 users.^116^

**Table 2.** Hormone replacement therapy (HRT) and breast cancer risk

	WHI		CGHFBC Meta-analysis		QResearch/CPRD		Million Women		*BRCA1* carriers	
	E alone	E + P	E alone	E + P	E alone	E + P	E alone	E + P	E alone	E + P
Study type	RCT		Prospective (24 studies) and retrospective (34 studies)		Retrospective (case control)		Prospective		Prospective (longitudinal cohort)	
HRT constituent	CEE (E); placebo	CEE (E) + MPA (P); placebo	CEE or E_2_ (E)	(E) + MPA, NETA, or LNG (P)	CEE or E_2_ (E)	(E) + NETA, LNG, MP, or DDG (P)	CEE or EE (E)	(E) + MPA, NET, NGT, or LNG (P)	N/A	N/A
HRT dose	0.625 mg/day	CEE, 0.625 mg/day; MPA, 2.5 mg/day	CEE, 0.3 to >0.625 mg/day; E_2_, 1–2 mg/day	N/A	CEE, ≤0.625 mg/d; E_2_, ≤1 mg/d; E_2_ gel, ≤50 mg	N/A	CEE, ≤ or > 0.625 mg/day; EE, ≤ or > 1 mg/day	N/A	N/A	N/A
HRT duration, years	7.2	5.6	<1 to ≥15 Mean, 10 (SD 6)		<1 to ≥10		<1 to ≥10		0.5 to 19 Mean, 3.9	
Participants	10 739; 5310 (E alone), 5429 (placebo)	16 608; 8506 (E + P), 8102 (placebo)	568 859 (total); 143 887 (BC cases), 424 972 (controls); 37 213 (E alone), 37 951 (E + P)		556 109 (total); 98 611 (BC cases), 457 498 (controls); 51 659 (E alone), 124 435 (E + P); 380 015 (No HRT)		828 923 (total); 115 383 (E alone), 142 870 (E + P), 392 757 (no HRT)		872 *BRCA1* carriers with BO (total); 377 (HRT), 495 (no HRT); 259 (E alone, 69%), 66 (E + P, 18%), 40 (P alone, 11%)	
Mean age (SD) at recruitment, years	63.6 (7.3)	63.2–63.3 (7.1)	55–72	50–67	Mean age (SD) at BC diagnosis: cases, 63.4 (8.3); controls, 63.3–63.6 (8.3)		55.9 age range, 50–64		HRT, mean 40.3 (range, 21–67); no HRT, 45.8 (21–74)	
Hysterectomy	Yes	No	Yes (84%)	No (93%)	Yes	No	N/A	N/A	N/A	N/A
Oophorectomy (BO)	4049 (37.7%)	53 (0.3%)	N/A	N/A	N/A	N/A	N/A	N/A	872 (100%)	
Follow-up, years	16.2–20.7		0–11	0–8	—	—	2.6–4.1		HRT, mean 6.2 (range 0.6–22); no HRT, 5.8 (0.1–18)	
BC risk^a^ overall	**0.78 (0.65–0.93) HR (95% CI)**	**1.28 (1.13–1.45)**	**1.17 (1.10–1.26) RR (95% CI)**	**1.60 (1.52–1.69)**	**1.06 (1.03–1.10) OR (95% CI)**	**1.26 (1.24–1.29)**	**1.30 (1.22–1.38) RR (95% CI)**	**2.00 (1.91–2.09)**	0.73 (0.41–1.32) HR (95% CI)	1.31 (0.66–2.57)
BC risk by age/HRT duration	0.77 (0.57–1.06) 50–59 years	**1.36 (1.09–1.69) 50–59 years**	**1.33 (1.19–1.48) 40–44 years**	**2.22 (1.96–2.52) 40–44 years**	1.08 (0.99–1.18) 50–59 years ≥5 years use	**1.57 (1.48–1.66) 50–59 years ≥5 years use**	0.81 (0.55–1.20) <1 year use	**1.45 (1.19–1.78) <1 year use**	0.47 (0.20–1.15) BO <45 years	1.64 (0.68–3.98) BO <45 years
	0.79 (0.61–1.02) 60–69 years	**1.22 (1.02–1.48) 60–69 years**	**1.39 (1.30–1.48) 45–49 years**	**2.14 (2.03–2.26) 45–49 years**	**1.17 (1.09–1.25) 60–69 years ≥5 years use**	**1.83 (1.75–1.91) 60–69 years ≥5 years use**	**1.25 (1.10–1.41) 1–4 years use**	**1.74 (1.60–1.89) 1–4 years use**	0.59 (0.25–1.40) BO <45 years; E alone or E + P ≤5 years use	
	0.76 (0.52–1.12) 70–79 years	1.27 (0.96–1.67) 70–79 years	**1.33 (1.25–1.42) 50–54 years**	**2.10 (2.01–2.21) 50–54 years**	**1.25 (1.11–1.39) 70–79 y ≥5 years use**	**2.20 (2.02–2.39) 70–79 years ≥5 years use**	**1.32 (1.20–1.46) 5–9 years use**	**2.17 (2.03–2.33) 5–9 years use**	**0.24 (0.06–0.98) BO <45 years; E alone or E + P >5 years use**	
	0.75 (0.54–1.03) E alone/BO All ages		**1.26 (1.12–1.41) 55–59 years**	**1.97 (1.81–2.15) 55–59 years**	**1.14 (1.08–1.21) all ages 5–9 years use**	**1.70 (1.64–1.76) all ages 5–9 years use**	**1.37 (1.22–1.54) ≥10 years use**	**2.31 (2.08–2.56) ≥10 years use**	**3.38 (1.17–9.73) BO <45 years; P alone or E + P ≤5 years use**	
	0.31 (0.06–1.45) E alone/BO 50–59 years		1.08 (0.90–1.31) 60–69 years	**1.75 (1.48–2.06) 60–69 years**	**1.17 (1.08–1.27) all ages ≥10 years use**	**2.05 (1.94–2.17) all ages ≥10 years use**			1.78 (0.18–17.7) BO <45 years; P alone or E + P >5 years use	
BC mortality (95% CI)	**0.60 (0.37–0.97)**	1.35 (0.94–1.95)	N/A	N/A	N/A	N/A	1.22 (1.00–1.48)		N/A	N/A
							**1.15**^b^ **(1.01–1.32) <5 years use**	**1.39**^b^ **(1.27–1.53) <5 years use**		
							**1.35**^b^ **(1.24–1.47) ≥5 years use**	**1.64**^b^ **(1.52–1.76) ≥5 years use**		
References	*JAMA*. 2020^44^ *Ann Intern Med*. 2019^135^		*Lancet*. 2019^40^		*BMJ*. 2020^60^		*Lancet*. 2003,^59^ 2019^61^		*JAMA Oncol*. 2018^137^	

Estrogen-alone HRT is highlighted in light pink; estrogen-plus-progestin HRT in light blue. Statistically significant risk is represented in boldface.

BC, breast cancer; BO, bilateral oophorectomy; CEE, conjugated equine estrogens; CGHFBC, Collaborative Group on Hormonal Factors in Breast Cancer; CI, confidence interval; CPRD, Clinical Practice Research Datalink; DDG, dydrogesterone; E_2_, estradiol; E, estrogen; EE, ethinylestradiol; HR, hazard ratio; LNG, levonorgestrel; MP, medroxyprogesterone; MPA, medroxyprogesterone acetate; N/A, information not available; NET, norethisterone; NETA, norethisterone acetate; NGT, norgestrel; OR, odds ratio; P, progestin; RCT, randomized clinical trial; RR, relative risk; SD, standard deviation; WHI, Women’s Health Initiative.

a BC risk estimated using incidence or cases of invasive breast cancer.

b BC mortality calculated with 20-year follow-up after recruitment.
